# Spatial variation and associated genes of total hair follicle density in goats

**DOI:** 10.5713/ab.25.0026

**Published:** 2025-05-12

**Authors:** Jipan Zhang, Ziyi Chen, Siyuan Zhang, Guoqiu Li, Xingqiang Fang, Yongju Zhao

**Affiliations:** 1 College of Animal Science and Technology, Southwest University, Chongqing, China; 2 Chongqing Key Laboratory of Herbivore Science, Chongqing, China; 3 Chongqing Engineering Research Center for Herbivores Resource Protection and Utilization, Chongqing, China; 4 College of Animal Sciences, Zhejiang University, Hangzhou, China

**Keywords:** Goat, Hair Follicle Density, Image Analysis, mRNA-Seq, Spatial Pattern

## Abstract

**Objective:**

Improving total hair follicle density (THFD) can directly result in increased fiber yield in cashmere goats. However, its phenotypic and genetic characteristics remain poorly understood. This study aimed to investigate the spatial distribution and associated genes of THFD in goats.

**Methods:**

A large-scale histological image analysis was conducted on 791 skin samples to characterize the spatial distribution of THFD at the local-site (n = 7), body-side (32 sites, n = 224), and whole-body (14 sites, n = 560) levels. Additionally, transcriptome sequencing was performed on 18 skin samples to identify genes associated with THFD.

**Results:**

The unique structure of the hair follicle group leads to an uneven distribution of hair follicles, resulting variation in THFD at the local-site. THFD exhibited a gradient pattern throughout the body, characterized by elevated values in the scapular region and decreased values in the abdominal region. THFD showed uneven distribution across the whole-body, with a consistent pattern observed in Inner Mongolia cashmere goats, Dazu black goats and their F1 hybrids (DBG♂ × IMCG♀). Through the integration of differential gene expression analysis, trend analysis, weighted correlation network analysis, Venn analysis, and literature review, four core genes (*GJA1*, *DSP*, *CDH3*, and *PER1*) were identified. These genes showed high and specific expression in skin tissue and demonstrated a significant response to changes in THFD.

**Conclusion:**

This study thoroughly characterized the spatial variation of THFD at the local-site, body-side, and whole-body levels in order to gain a better understanding of genetic characterization, associated genes and spatial variation. Additionally, we have identified four genes that are associated with THFD. These findings provide an important reference for the subsequent development and utilization of THFD genes in cashmere goats.

## INTRODUCTION

Cashmere fiber, often referred to as “soft gold,” is a high-grade textile material that has considerable economic value [1]. Certain goat breeds, such as the Inner Mongolian cashmere goat (IMCG) and Liaoning Cashmere goat, are famous breeds specifically for cashmere production [2,3]. Cashmere goats have double coats, with coarse hair produced by primary hair follicles for mechanical protection and cashmere produced by secondary hair follicles for thermal insulation [4]. The fleece yield is greatly influenced by fiber density [5], which is commonly measured as total hair follicle density (THFD) during histological examinations [6]. THFD is defined as the total number of hair follicles per unit area of the skin staining section. Studies have shown that animals with higher THFD such as goats [7], sheep [8], and Huacaya alpacas [9] tend to have finer fiber diameters. Breeding animals with smaller fiber diameters, longer fiber lengths, and higher fiber densities is a long-term objective. The selection of goats with high THFD, therefore, holds significant importance in the production of fur fiber animals.

Fiber density is influenced by various factors, including genetic traits such as breed [10] and nongenetic factors such as developmental stage [5], melatonin treatment [11], and nutritional levels [12]. Of note, most hair follicles develop during the prenatal stage, with few additional follicles forming after birth [13], which implies that fiber density in adult goats largely depends on the hair follicle reserve established at birth. As goats become adults, their rapidly increasing trunk surface area directly reduces fiber density. Although the temporal development of hair follicles from the embryonic to adult stages and the annual follicle cycle [14,15] have been studied in detail, there is limited knowledge regarding the spatial distribution of THFD.

The RNA sequencing (RNA-seq) technique provides an effective method to identify important functional gene groups under different experimental conditions [16]. Studies have successfully applied this approach to explore genetic mechanisms underlying fiber density or follicle density in animals. For example, Wang et al. identified *FOXM1* and *CDK1* related to feather follicle density in Wannan chickens [17] and Jiang et al found a mutation in *MAP2* linked to hair follicle density in pigs [18]. In addition, Ding et al. identified five long noncoding RNAs between high and low hair follicle density groups in Angora rabbits [19]. However, there is limited knowledge regarding THFD-associated core genes in goats.

In the present study, a large-scale histological investigation was conducted to explore THFD distribution patterns across the local-site, body-side, and whole-body levels. In addition, 18 skin transcriptome data were obtained to identify candidate genes associated with THFD.

## MATERIALS AND METHODS

### Animals, samples, and experimental design

This study was conducted as per the guidelines set by the Animal Care and Use Committee of Southwest University (No. IACUC-20230417-02). A total of 47 adult goats, including 18 IMCGs, 9 Dazu black goats (DBGs), and 20 F1 hybrids (DBG♂ × IMCG♀), were included in this study. As shown in Supplement 1, the F1 hybrids were produced via the crossbreeding of female IMCGs and male DBGs. All goats were raised at the Southwest University farm and fed a diet comprising alfalfa, barley grain, and wheat straw, satisfying the National Research Council’s requirements for goat maintenance. This study was divided into three parts.

#### Part I

**Figure 1:**
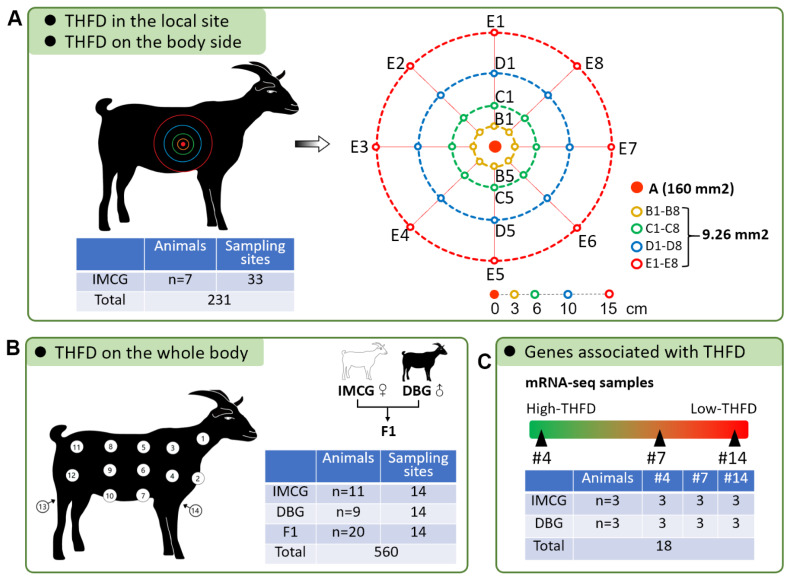
Experimental design. (A) Skin tissues of seven IMCGs were collected from 33 sites on the right side of the body. The central sampling site on the posterior scapula (#6) was used to make a large histological section to investigate spatial variation of THFD in the local site (physical area of 160 mm^2^). Besides, another 32 sites were used to analyze spatial variation of THFD on the body side (area of 7,000 mm^2^). (B) IMCGs and DBGs are typical goat breeds that produce cashmere and meat, respectively. Female IMCGs and male DBGs were mated, and their F1 hybrids were raised to the adult stage. Skin tissues were collected from 14 sites in the whole body of 11 IMCGs, 9 DBGs, and 20 F1 hybrids (DBG♂ × IMCG♀). These samples were used to analyze spatial variation of THFD in the whole body and observe phenotypic changes due to genetic changes. (C) Based on the THFD distribution pattern on the whole body, three sites (#4, #7, and #14) were observed to be close and with large THFD changes. Finally, 18 skin tissues were collected to perform mRNA-seq and investigate THFD-associated genes. THFD, total hair follicle density; IMCG, Inner Mongolia cashmere goat; DBG, Dazu black goat.

Seven female IMCGs (age, 1.5 to 2 years) were slaughtered by cutting their jugular vein. First, the skin fibers of the slaughtered goats were removed using an electric hair clipper (FC5902; Flyco, Shanghai, China) in a 5×5 cm area around 33 designated points on the right side of the body (Figure 1A). A total of 231 skin tissue samples were collected using a surgical scissor and stored in a 4% paraformaldehyde fixation solution. These samples were eventually used for histological image analysis to investigate spatial variation of THFD at both the local-site and body-side levels.

#### Part II

Forty adult goats, including 11 IMCGs (6 females and 5 males; age, 1.5 to 2 years), 9 DBGs (5 females and 4 males; age, 1.5 to 2 years), and 20 F1 hybrids (5 females and 15 males; age, 1.5 to 2 years), were selected and slaughtered by cutting their jugular vein. Next, 560 skin tissue samples were collected from 14 body sites from each animal (Figure 1B) and stored in a 4% paraformaldehyde fixation solution. Please note that the samples belonging to 11 IMCGs were derived from our previous study [20] but would be subjected to in-depth analysis in this study. These samples were used to analyze spatial variation of THFD at the whole-body level and observe phenotypic changes due to genetic changes.

#### Part III

During the collection of samples in experimental part II, additional skin samples were retained from three IMCGs and three DBGs and stored in an RNA/DNA protection reagent (CWBIO, Jiangsu, China) at −80°C (Figure 1B). Of these, 18 skin samples (belonging to sites #4, #7, and #14) were finally used for transcriptome analysis to explore genes related to hair follicle and fleece traits.

### Skin section, hematoxylin and eosin staining, and image analysis

After 48 h of fixation, the skin samples were dehydrated in an alcohol gradient, cleared in xylene, and embedded in paraffin in the form of transverse sections. Their skin surfaces were placed uppermost and level parallel to the wax block. The paraffin-embedded samples were sectioned at a thickness of 5 μm using a rotary microtome (RM2235; Leica, Wetzlar, Germany). Finally, clear and intact sections at the sebaceous gland depth level were selected for HE staining, and a 40X image was captured for every stained section using a microscope camera (Olympus, Tokyo, Japan). For all samples, except for the seven samples belonging to the center point in experimental part I, only one image with a physical area of 9.26 mm^2^ was retained and analyzed. For each of the seven center samples, the images were merged using the photomerge function in the Photoshop software (Adobe, San Jose, CA, USA) and then cropped to a physical size of 160 mm^2^.

All secondary hair follicles were manually marked as blue dots, whereas primary hair follicles were marked as green dots using the Image-Pro Plus software (Media Cybernetics, Rockville, MD, USA). The hair follicle group (HFG) regions were manually marked as color blocks using the lasso tool in Photoshop (Adobe). Next, a self-written Matlab script was used to extract the marked color dots and blocks. The following hair follicle traits were calculated: (1) THFD, the number of total hair follicles divided by the entire image area; (2) S/P, the ratio of secondary and primary hair follicles; (3) Ratio-HFG, the ratio of HFG-occupied regions to the entire image area; and (4) Int-THFD, THFD in HFG-occupied regions. For every sample, the following formula always holds: THFD = Int-THFD×Ratio-HFG.

### Spatial variation of total hair follicle density

#### (1) THFD in the local site

The seven images (each of 160 mm^2^) were divided into 4, 9, 16, and 25 equal parts, and their corresponding THFD was compared. The heatmap of THFD values was visualized using the self-written Matlab script.

#### (2) THFD on the body side

To explore THFD variation on the body side, hair follicle traits from 32 samples were compared.

#### (3) THFD in the whole body

THFD values were compared based on the 14 body sites distributed on the whole body. Besides, THFD patterns on IMCG, DBGs, and their hybrids were compared. The comparison was performed using one-way analysis of variance.

### Skin transcriptome sequencing

Sites #4, #7, and #14 were close to each other but had large THFD differences. Therefore, 18 skin samples were selected from three sites on six goats (three IMCGs and three DBGs; Figure 1C) and sent to Biomarker Technologies (www.biomarker.com.cn) for mRNA extraction, library construction, and mRNA seq on the Illumina HiSeq 4000 platform. Adapter sequences, low-quality reads, and reads with poly-N were removed using the Trimmomatic software [21], and the Q20, Q30, GC-content, and sequence duplication levels of the clean data were calculated. In addition, the STAR software [22] was used to map clean reads to the reference genome (ASR1.2, [23]) and the StringTie [24] software was used to quantify mRNA expression level as per fragments per kilobase million (FPKM).

### Determination of fleece traits

Considering that the skin tissue serves as the carrier of hair fibers and bulk mRNA-seq reflects the expression profile of the entire tissue, we also measured the fleece traits corresponding to skin samples from sites #4, #7, and #14. The fleece traits include (1) fleece melanin content, (2) hair length, (3) cashmere length, (4) hair diameter, and (5) cashmere diameter. The fleece melanin content was measured using the NaOH assay method. In brief, fleece samples were washed with ethanol and cut into 1 to 2-mm lengths. Then, 10 to 30 mg of each fleece sample was placed in a 5ml centrifuge tube containing 2 mL of 1 mol/L NaOH and water bathed at 95°C for 1 h. Absorbance was measured at 500 nm using a microplate reader (Bio-rad, Hercules, CA, USA), and a standard working curve was established based on the absorbance values obtained from the different concentrations of standard melanin (Aladdin, Shanghai, China). The fleece melanin content was calculated using its optical density value and the standard curve. The cashmere length and hair length were measured using a steel ruler, with each trait measured three times for each sample. The above-mentioned cut fleece sample was taken and mixed with a drop of glycerol on a glass slide and then covered with a coverslip. The glass slide was kept under the inverted microscope, and cashmere diameter and hair diameter were recorded using the Cellsens software. The cashmere diameter and hair diameter were measured at least 30 times for each sample.

### Differentially expressed gene analysis and trend analysis

Principal component analysis (PCA) was used to describe global transcriptomic differences between samples. In addition, hierarchical clustering and a heatmap of the top 1,000 genes with the largest variance were generated using the heatmap R package. Differentially expressed gene (DEG) analysis was performed on all IMCG and DBG samples. The criteria for identifying DEGs included a p-value of <0.01 and a fold change of >2. DEG analysis was also performed between sites belonging to a single goat breed. Meanwhile, trend analysis was performed to identify intersecting genes with similar expression patterns between IMCGs and DBGs. Based on genes identified using DEG and trend analyses, the Kyoto Encyclopedia of Genes and Genomes (KEGG) analysis was performed using the OmicShare tool (https://www.omicshare.com/tools).

### Weighted gene coexpression network analysis

In the current study, weighted gene coexpression network analysis (WGCNA) was performed using the SangerBox platform (http://www.sangerbox.com/). Correlation coefficients between the gene module and traits (THFD and fleece color) were calculated, and key modules with p<0.001 and R>0.6 were retained. Next, the gene module membership was calculated in the hub module, which was then used to measure the importance of each gene in the module. Hub genes were defined as genes with the highest degree of connectivity in the significant module. All hub genes were included in KEGG analysis using the OmicShare tool.

### Venn analysis and quantitative polymerase chain reaction validation

Nineteen genes were randomly selected for quantitative polymerase chain reaction (qPCR) experiments to validate their expression. *ACTB* and *GAPDH* were set as double reference genes. The paired primers of all 21 genes are listed in Table 1. mRNA extraction, reverse transcription, qPCR, amplification specificity, and relative gene expression analysis were performed as described earlier [25]. Genes identified using three approaches (DEGs, trend analysis, and WGCNA) were further analyzed using Venn analysis. Based on the Web of Science, we carefully reviewed the related publications for every one of the overlapped genes. Genes that have established functions in the field of “skin”/“hair” biology, supported by literature, were retained.

### Tissue expression profile and gene-trait correlation

To investigate whether the core genes exhibit specific expression in skin tissue, we downloaded 138 mRNA-seq samples. Seven tissues (heart, n = 13; liver, n = 20; spleen, n = 13; lung, n = 22; kidney, n = 20; muscle, n = 34; and skin, n = 16) were obtained from five projects (PRJEB42490, PRJEB23196, PRJNA160149, PRJNA309284, and PRJNA276799) on the NCBI database. Reads mapping and gene quantitation were performed as mentioned before. Additionally, based on the transcriptomic data of the 18 samples from this study, we explored the correlation between the expression levels of these core genes and the THFD trait.

## RESULTS

### Spatial variation of total hair follicle density in the local site

**Figure 2:**
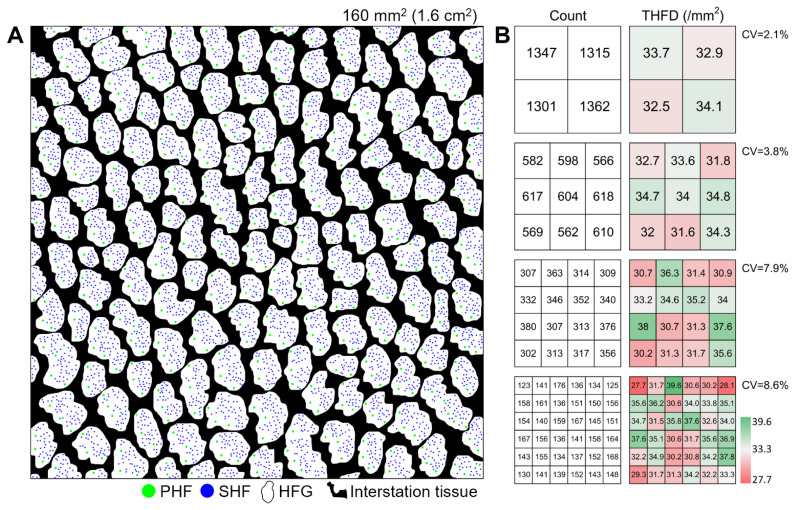
Spatial variation of THFD in the local site. (A) In the final clean image, the green/blue, white, and black represent the hair follicles, hair follicle groups, and interstitial tissue, respectively. (B) Counts and heatmap of TFHD values when the full image was divided into 4, 9, 16, and 25 subimages. With the decrease in the statistical area, the coefficient of variation between THFD values increased. THFD, total hair follicle density; CV, coefficient of variation; PHF, primary hair follicle; SHF, secondary hair follicle; HFG, hair follicle group.

After marking the hair follicles as color dots and HFGs as color blocks, computer-recognizable clean images were obtained (Figure 2A). After a sample was divided into 4, 9, 16, and 25 sub-images, the coefficient of variation kept increasing (from 2.1% to 8.6%; Figure 2B). The smaller the statistical area, the more easily THFD is affected by the skin structure. Even in a local site, THFD was not evenly distributed because of the following factors: (1) the presence of HFGs and (2) size of the statistical area.

### Spatial variation of total hair follicle density on the body side

**Figure 3:**
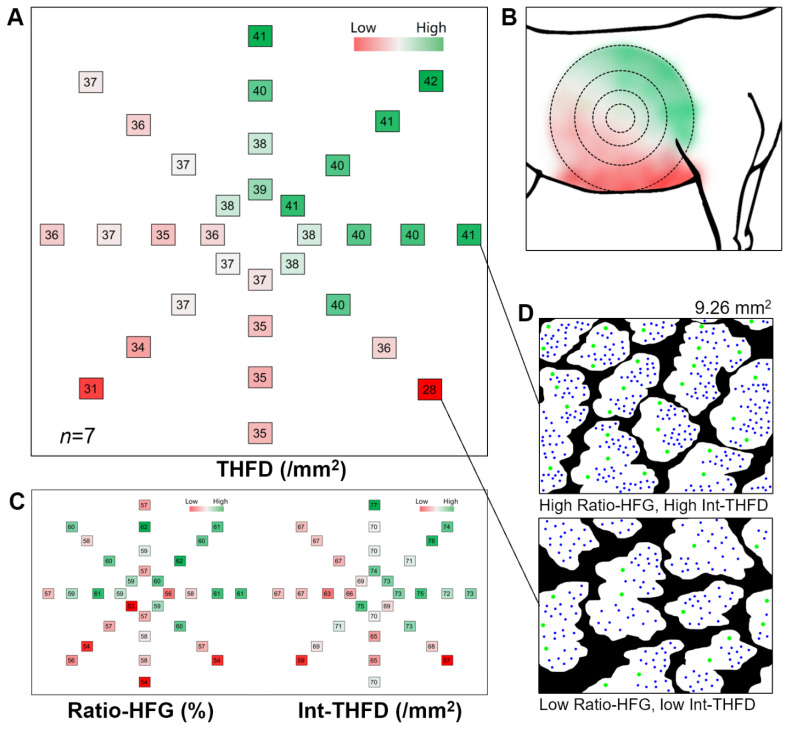
Spatial variation of THFD on the body side. (A) THFD heatmap of 32 sampling sites on the body side. (B) The simulation diagram of THFD was based on the hypothesis of gradient changes among 32 sampling sites. (C) Heatmap of the Ratio-HFG and Int-THFD of the 32 sampling sites. (D) Image comparison between two representative sites with significant THFD changes. THFD, total hair follicle density; HFG, hair follicle group; Ratio-HFG, the ratio of HFG-occupied regions to the entire image area; Int-THFD, THFD in HFG-occupied regions.

The 32 sampling sites on the body side had circular radiological distribution. In the image, the high THFD region is on the upper right and the low value is on the lower right (Figure 3A). The rendering heatmap revealed the overall change in THFD value (Figure 3B). Meanwhile, the clean images on two adjacent samples showed a large difference in THFD (Figure 3C). The Ratio-HFG and Int-THFD were not evenly distributed, with larger values in the upper right (Figure 3D). Thus, they were identified as direct factors that affect the THFD value, indicating that the following formula was true for every sample: THFD = Ratio-HFG×Int-THFD.

### Spatial variation of total hair follicle density on the whole body

**Figure 4:**
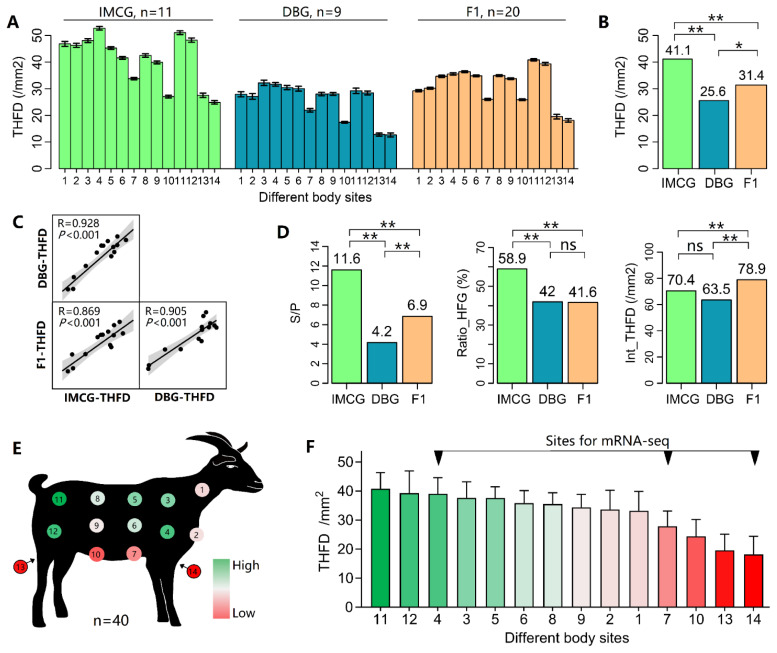
Spatial variation of THFD in the whole body. (A) Comparisons of THFD values on sites #1-#14 among IMCGs, DBGs, and their F1 hybrids. (B) Average THFD value of all 14 sites in IMCGs, DBGs, and their F1 hybrids. (C) Correlation analysis of THFD values among IMCGs, DBGs, and their F1 hybrids. (D) Average S/P, Ratio-HFG, and Int-THFD values of all 14 sites in IMCGs, DBGs, and their F1 hybrids. (E) Heatmap of the average THFD values of all goats (n = 40). (F) The 14 body sites were sorted as per the THFD value, ultimately selecting sites #4, #7, and #14 for mRNA-seq. IMCG, Inner Mongolia cashmere goat; DBG Dazu black goat; F1, DBG♂×IMCG♀; THFD, total hair follicle density; Ratio-HFG, the ratio of HFG-occupied regions to the entire image area; Int-THFD, THFD in HFG-occupied regions.

Among the 14 body sites, the lowest two THFD values consistently appeared in sites #13 and #14 (Figure 4A). As a cashmere-producing goat breed, IMCGs exhibited a higher THFD than DBGs and their F1 hybrids (p<0.01, Figure 4B), but their patterns of THFD distribution were highly consistent (Figure 4C). IMCGs had an S/P value of 11.6, about three times that of DBGs and twice that of F1 hybrids. The Ratio-HFG of IMCGs was 58.9%, approximately 1.5 times that of DBGs (41%) and F1 hybrids (41.2%). The Int-THFD values of IMCGs, DBGs, and F1 hybrids, respectively, were 70.4, 63.5, and 78.9 hair follicles/mm^2^ (Figure 4D). Of note, the difference in Ratio-HFG is the main factor that directly determines the difference in THFD. Figure 4E shows the THFD distribution in the whole body, and high THFD was observed in the front while low THFD was detected in the bottom of the body.

### Fleece traits in three body sites

**Figure 5:**
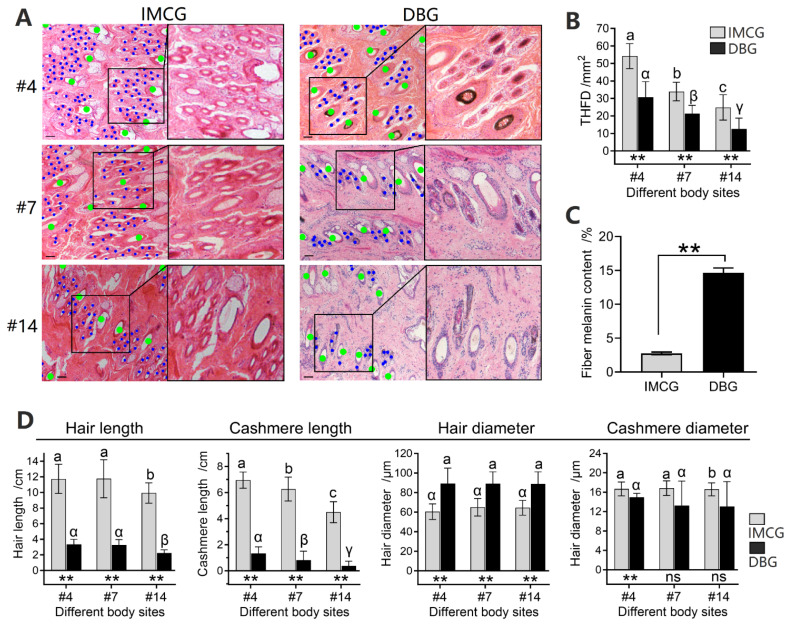
Histological image and THFD in sites #4, #7, and #14. (A) Representative images of sites #4, #7, and #14 in IMCGs and DBGs. (B) THFD varies greatly among the three body sites. Green and blue dots represent primary and secondary hair follicles, respectively. (C) The comparison of fiber melanin content between IMCGs and DBGs. (D) The comparison of fiber traits among three body sites in IMCGs and DBGs. a–c Significant differences among the sites in IMCG (p<0.05). α–γ Significant differences among the sites in DBG (p<0.05). ** represent significant differences between IMCG and DBG at the same body site. IMCG, Inner Mongolia cashmere goat; DBG Dazu black goat; THFD, total hair follicle density.

Sites #4, #7, and #14 were adjacent sites with significant THFD differences (Figures 5A, 5B). Considering that bulk transcriptome sequencing was performed on these skin samples, the fleece traits were also measured. The IMCG and DBG exhibit solid white and solid black colors, respectively; thus their fiber melanin content was significantly different (p<0.01, Figure 5C). As a famous cashmere goat breed, IMCG possesses hair length of up to 10 cm, which is significantly longer than that of DBG (p<0.01, Figure 5D). The cashmere lengths measured at three sites (#4, #7, and #14) in IMCG are 7.0±0.62, 6.3±0.91, and 4.5±0.81 cm, respectively, which are significantly longer than those of DBG, corresponding to 1.3±0.51, 0.8±0.68, and 0.4±0.36 cm (Figure 5D). Moreover, DBG exhibits thicker coarse hairs and finer cashmere than IMCG, but no significant differences were observed in the three measured sites. Overall, significant differences are not only observed between IMCG and DBG for traits such as THFD, hair length, and cashmere length, but also among different body sites (#4, #7, and #14). Therefore, THFD and three additional traits, including fiber melanin content, hair length, and cashmere length were explored for gene expression profile in subsequent transcriptome analysis.

### DEG analysis and trend analysis

The transcriptome sequence of the 18 skin samples produced 148.8 Gb of raw data (Table 2). After performing quality control, reads mapping, transcript assembly, and gene quantification, an FPKM matrix containing 15,434 genes across 18 samples was obtained.

**Figure 6:**
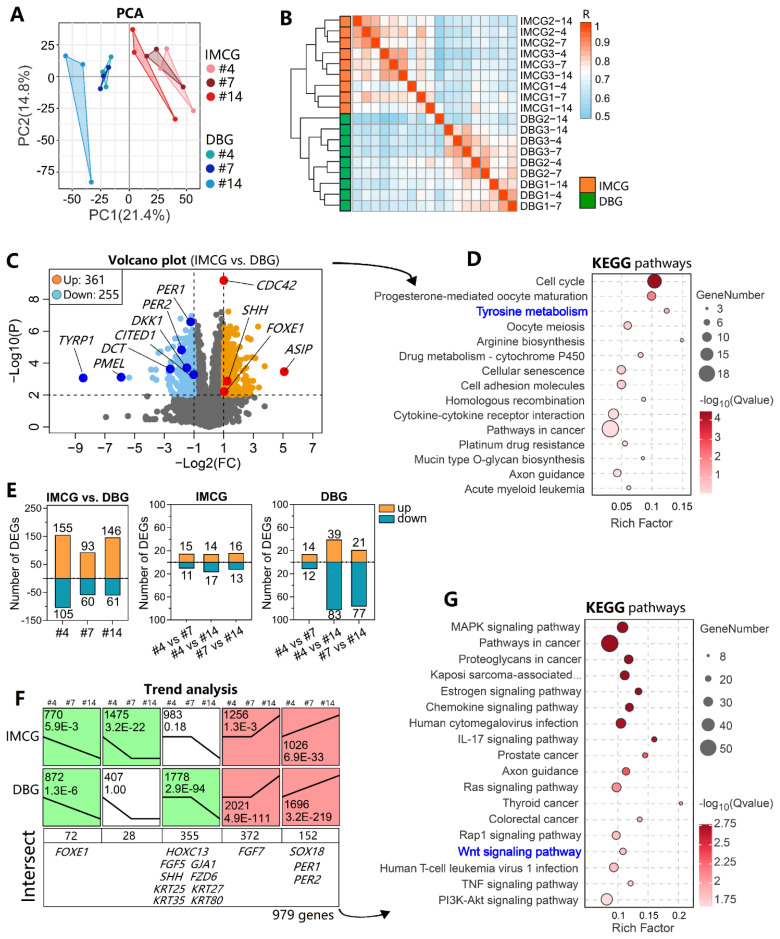
DEG analysis and trend analysis. (A) PCA score plot showing inter-group differences. (B) Hierarchical clustering heatmap of top 1,000 genes with the largest variance across 18 samples. (C) Volcano plot of DEGs between IMCGs and DBGs. (D) KEGG enrichment pathways based on DEGs between IMCGs and DBGs. (E) Statistical analysis of DEGs between different body sites. (F) Count of genes and trend types following trend analysis. (G) KEGG enrichment based on 979 genes identified via trend analysis. PCA, principal component analysis; IMCG, Inner Mongolia cashmere goat; DBG Dazu black goat; KEGG, the Kyoto Encyclopedia of Genes and Genomes; DEG, differentially expressed gene.

The PCA plot (Figure 6A) and heatmap (Figure 6B) of the top 1,000 highly variable genes displayed the overall differences between IMCGs and DBGs. A total of 339 upregulated and 224 downregulated DEGs were identified between IMCGs and DBGs (Figure 6C). Of these, hair follicle-related genes such as *DKK1*, and *SHH*, melanogenesis genes such as *ASIP*, *DCT*, *PMEL*, and *TYRP1*, and biological rhythm-associated genes such as *PER1* and *PER2* were identified. Based on these DEGs, KEGG pathway enrichment was performed, which identified “tyrosine metabolism” (Figure 6D), possibly because of the huge difference in coat color between IMCGs and DBGs. When the three sites (#4, #7, and #14) in both IMCGs and DBGs were compared, only dozens of DEGs were identified (Figure 6E), probably owing to the limited sample size. The union of DEGs identified under various strategies comprises a total of 725 genes.

The three sites were combined into eight trend types, and five trend types were significant in the two goat types. Genes with similar trends were reviewed in both IMCGs and DBGs, and 72, 28, 355, 372, and 152 genes were identified after performing intersect functions (Figure 6F). Among these genes, *FOXE1, HOXC13, GJA1, FGF5, FGF7, SHH, KRT25*, and *KRT27* were noted. In addition, based on the 979 genes identified using trend analysis, the “MAPK signaling pathway” and “Wnt signaling pathway” were significantly enriched (Figure 6G).

### Weighted correlation network analysis

**Figure 7:**
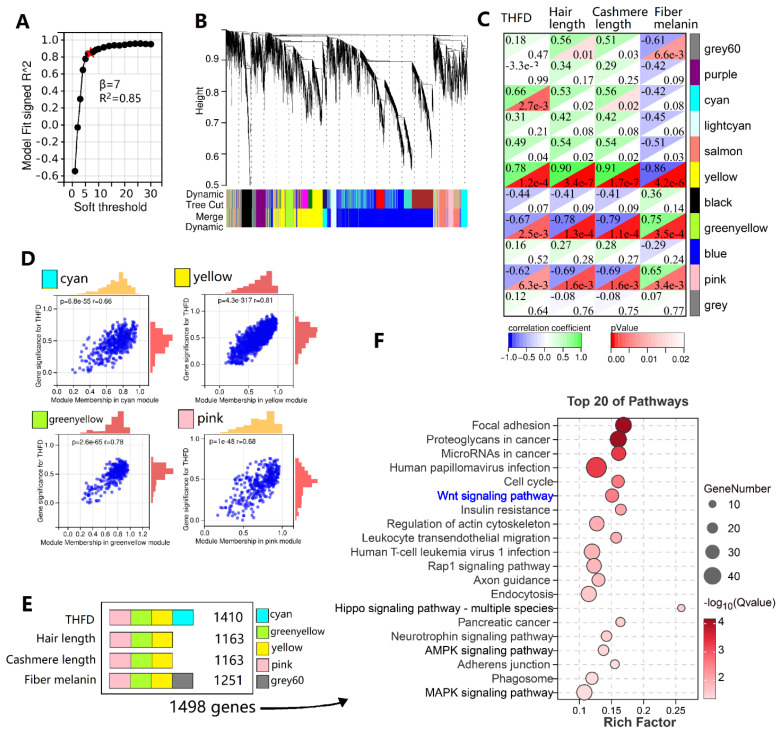
Weighted gene coexpression network analysis. (A) Determination of the soft threshold for adjacency matrix. (B) Hierarchical clustering and key modules identified using WGCNA. (C) Correlation coefficients between key modules and traits. (D) Scatterplots of gene significance vs. module membership for THFD traits. (E) Schematic diagram showing the counted number of hub genes in key modules. (F) KEGG enrichment pathways based on 1,498 hub genes identified using WGCNA. THFD, total hair follicle density; KEGG, the Kyoto Encyclopedia of Genes and Genomes; WGCNA, weighted gene coexpression network analysis.

WGCNA was performed to explore genes associated with hair follicle or fleece traits. The soft-threshold parameter (β) was set to 7 (Figure 7A), which clustered 14,929 genes in 11 major coexpression modules (Figure 7B). According to the module-trait association, the “yellow,” “green-yellow,” “pink,” “cyan,” and “grey60” modules were selected as they were significantly associated (R>0.6, p<0.01) with THFD, hair length, cshmere length, and fleece color (Figure 7C). For THFD, the scatterplots showed a highly significant correlation between gene significance and module membership (Figure 7D). The five modules, respectively, comprised 246, 196, 220, 747, and 89 genes, totalling 1498 genes (Figure 7E). KEGG identified the “Wnt signaling pathway,” “Hippo signaling pathway,” “AMPK signaling pathway,” and “MAPK signaling pathway” with these genes (Figure 7F). These pathways are reportedly associated with hair follicle development and the hair follicle cycle.

### Quantitative polymerase chain reaction validation and Venn analysis

**Figure 8:**
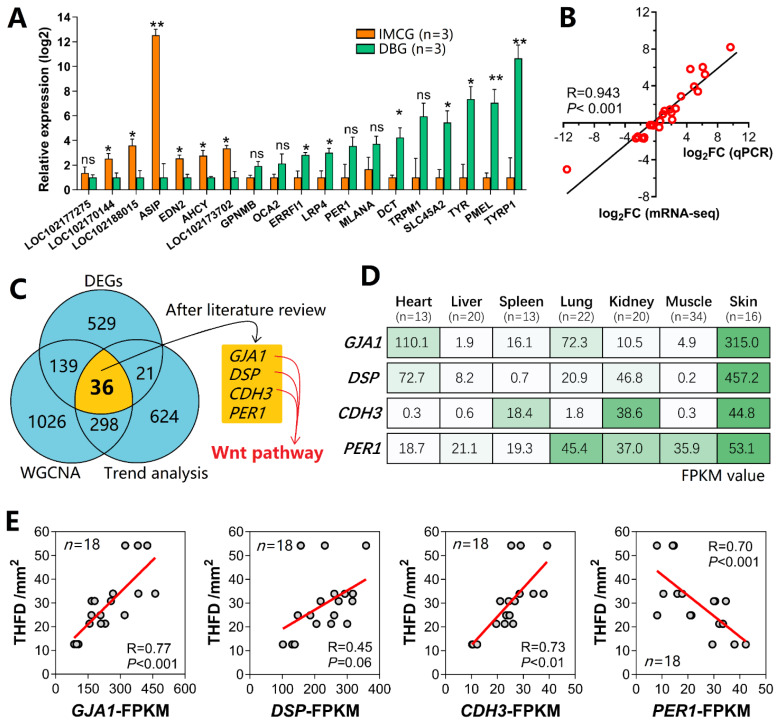
qPCR validation and Venn analysis. (A) Relative expression levels of 19 target genes in IMCGs (site #4, n = 3) and DBGs (site #4, n = 3) determined by qPCR experiments. (B) Correlation analysis of gene expression between qPCR and mRNA-seq results, with the linear trend line and correlation coefficient shown. (C) Venn diagram identified 36 genes overlapped in DEG analysis, trend analysis, and WGCNA. After conducting a literature review in the Web of Science, four genes (*GJA1*, *DSP*, *CDH3*, and *PER1*) were supported by literature that plays a crucial role in skin biology. (D) The expression profiles of the four core genes in the heart, liver, spleen, lung, kidney, muscle, and skin tissues. The highly and specifically expressed in skin tissue of these four genes indicated a certain degree of tissue-specific expression. (E) The correlation scatter plots between target genes (*GJA1*, *DSP*, *CDH3*, and *PER1*) and THFD trait. * p<0.05, ** p<0.01. IMCG, Inner Mongolia cashmere goat; DBG, Dazu black goat; qPCR, quantitative polymerase chain reaction; DEG, differentially expressed gene; WGCNA, weighted gene coexpression network analysis; FPKM, fragments per kilobase million; THFD, total hair follicle density.

For evaluating gene expression profiles, 19 genes were randomly selected (Figure 8A). The melanin-related genes of *ASIP, EDN2, AHCY, OCA2, DCT, SLC45A2, TYR, PMEL*, and *TYRP1* were significantly differentially expressed between IMCGs and DBGs. The comparison between mRNA-seq and qPCR data validated the accuracy and consistency of the mRNA-seq data (Figure 8B). The three approaches of DEG analysis, trend analysis, and WGCNA, respectively, identified 725, 979, and 1498 genes associated with hair follicle or fleece traits. As shown in the Venn diagram, 36 genes overlapped the three approaches (Figure 8C). After conducting a literature review based on the Web of Science, four genes (*GJA1*, *DSP*, *CDH3*, and *PER1*) were found they have a crucial role in skin biology. Interestingly, *GJA1*, *DSP*, and *CDH3* were involved in the Wnt signaling pathway.

### Multiple tissue expression profile and gene-trait correlation

Based on the FPKM matrix derived from 138 multi-tissue transcriptomic samples, we examined the expression profiles of *GJA1*, *DSP*, *CDH3*, and *PER1* genes. Excitingly, these genes were found to be highly and specifically expressed in skin tissue (Figure 8D), which to some extent indicates that they play important roles in hair follicle biology. Correlation analysis between gene expression and the THFD trait revealed a strong association (Figure 8E). Specifically, genes *GJA1*, *DSP*, and *CDH3* exhibited positive correlations with THFD, whereas *PER1* showed a negative correlation. Notably, *GJA1* had the highest correlation with the THFD trait (R=0.77, p<0.001). These findings suggest that the expression levels of these four core genes were responsive to variations in THFD.

## DISCUSSION

In this study, we investigated spatial variations in goat THFD at the local-site, body-side, and whole-body levels, and also identified genes related to THFD based on spatial patterns and breed differences. These findings provide new insights into the understanding of THFD from both the phenotypic and transcriptomic perspectives.

As the study focused on hair follicle density, histological investigation was unavoidable. For this, 7 ultra-large transverse skin sections, each covering an area of 160 mm^2^ and containing over 5,000 hair follicles, were prepared. To our knowledge, this is the largest and most intact transverse image in the field of skin research on fur fiber animals. While there is a consensus that a section should be kept at the level of the sebaceous gland [6], ensuring that all hair follicles are within a single transverse section is difficult. One major challenge encountered during the experimental process was skin wrinkles. To address this, the skin sample was vertically pressed using a flat spatula during the paraffin-embedding process. This method prevented horizontal deformation and simultaneously ensured that the skin surface was almost on the same plane.

One motivation for this study was to understand how THFD is distributed, from the local to the whole body. Goats possess a unique and independent skin histological structure, HFG, which is composed of several primary hair follicles, dozens of secondary hair follicles, corresponding sebaceous glands and sweat glands. The structure is relatively independent and occupies about 50% to 60% of the skin surface [26]. Its presence directly leads to an uneven distribution of hair follicles, thereby resulting in THFD variation in the local site. Even within hair follicle clusters, follicle distribution is uneven, with several primary follicles on one side and dozens of secondary follicles on the other. Thus, the spatial variation of THFD in the local site originates from two factors: (1) the uneven distribution of hair follicles within HFGs and (2) the uneven distribution of the HFGs in the skin.

The spatial variations of THFD in the body side exhibited a gradient pattern, with higher values at the scapular region and lower values at the abdominal region. Given that the hair typically grows downward (in alignment with gravity), the higher THFD in the upper body may partially compensate for the lower body regions. The spatial variation of THFD across the whole body exhibited a consistent pattern in cashmere goats, non-cashmere goats, and their F1 hybrids. This pattern may arise from the uneven growth during the organism’s development, as the increase in body weight and skin surface area directly leads to a reduction in hair density [27]. However, due to the lack of skin samples from goat neonates, it remains unknown whether the uneven distribution of THFD primarily originates from the developmental stage (the last two months of embryogenesis) or the postnatal growth stage (kids to adults). Additionally, the THFD at site #6 consistently remains close to the mean THFD on the body-side or whole-body, indicating that site #6 is a highly representative sampling site for population genetics studies of THFD in goats.

Another motivation for this study was to identify genes closely associated with THFD based on the transcriptomic data from skin samples with varying THFD across different body sites. Using multiple strategies, four core genes including *GJA1*, *DSP*, *CDH3*, and *PER1* genes had a significant response to changes in THFD were identified. *GJA1*, also known as connexin 43, is a crucial gap junction protein involved in intercellular communication. Most research on GJA1 has focused on human dermatological disorders, such as hypotrichosis (a skin disease characterized by hair loss) [28] and curly hair [29]. The *DSP* gene encodes desmoplakin, a desmosomal component found in the skin and heart, which provides structural integrity by connecting cells and anchoring intermediate filaments. Pathogenic mutations in the *DSP* gene can lead to a variety of phenotypes, including skin fragility and woolly hair in both humans [30] and mice [31]. *CDH3*, which encodes P-cadherin, is essential for skin development and mutations in this gene can cause hypotrichosis [32]. *PER1* (Period circadian regulator 1) is a well-known clock gene that plays a crucial role in regulating the hair cycle. The cycle of hair follicles, which includes anagen, catagen, and telogen phases, is strictly regulated by the circadian clock. Studies have shown that silencing the *PER1* gene can prolong the anagen phase in cultured hair follicles, highlighting the importance of the molecular clock in regulating the hair cycle [33]. In summary, these genes have been extensively studied and have been found to play significant roles in skin development and the hair cycle. Although no studies have directly reported associations between these genes and hair density or hair follicle density, evidence from research on hypotrichosis implies such connections. Goat is a species that facilitates the convenient collection of large numbers of skin samples. Therefore, further investigation into the mechanisms by which they regulate hair follicle density in goats will not only benefit the fur-fiber industry but also potentially contribute new insights to human dermatology.

It is important to recognize that the so-called “hair follicle density” genes identified in this study may not directly determine the formation of hair follicle density. As mentioned before, THFD in adult goats is primarily determined by the hair follicle reserve at birth [13]. Thus, the identified “hair follicle density formation” genes may be considered when conducting research in the late embryonic stage. Although “THFD” is typically defined as the number of hair follicles per unit area, it is influenced by various factors. Consequently, molecular mechanisms underlying the follicle-intensive development and hair follicle placode formation should be investigated concurrently.

Fiber color, a product of hair follicle activity, is an important characteristic that should not be ignored. In the current study, several genes, including *ASIP* and *AHCY*, along with pathways such as “tyrosine metabolism,” were significantly associated with melanogenesis. These genes have been implicated in numerous studies on animal coat color. For example, *ASIP* is a well-known gene that influences skin pigmentation and coat color in animals [34]; *AHCY* has also been reported to influence coat color in goats [35]. This finding is unsurprising, given the stark differences in coat color between DBGs and IMCGs.

## CONCLUSION

Through a large-scale histological investigation of 791 skin samples and transcriptome sequencing of 18 skin samples, we examined the spatial variation and genes associated with THFD in goats. The unique tissue structure of the HFG leads to THFD variation at the local-site; THFD exhibited a gradient pattern on the body-side; THFD unevenly distributed across the whole-body in cashmere goats, non-cashmere goats and their F1 hybrids. The transcriptome analysis identified four core genes (*GJA1*, *DSP*, *CDH3*, and *PER1*) and the Wnt signaling pathway that significantly associated with THFD. Our findings offer a significant reference for further elucidating the genetic determinants of hair density in cashmere goats.

## Figures and Tables

**Table 1:** Primer sequences

No.	Gene name	Primer sequence (forward/reverse)	Product size	°C^1)^
1	*ACTB* ^2)^	TGATATTGCTGCGCTCGTGGT	189	62.76
		GTCAGGATGCCTCTCTTGCTC	189	60.47
2	*GAPDH* ^2)^	TTATGACCACTGTCCACGCC	216	60.04
		TCAGATCCACAACGGACACG	216	60.04
3	*TYRP1*	TCAGTTTGTCATCGCCACCA	192	59.89
		AGAAATGCTGGTCCCTCGTG	192	60.04
4	*TRPM1*	TGAAGAGGCTGCACGAGTTT	108	59.89
		TCTCGCAGGTTACACGGATG	108	59.83
5	*SLC45A2*	GGATTCATCGGGCTCTTCCC	106	60.25
		CAGCGATGAGGGTAAAGGGT	106	59.46
6	*PMEL*	CTGGCTTGGTGTTTCAAGGC	255	59.97
		CACACCTGGCTCCCATTGAT	255	60.03
7	*MLANA*	AAAGGGCACAGCCACTCTTA	126	59.23
		GCTTCGGTATCCACTTCGTCT	126	59.87
8	*LOC102188015*	ACCTTCTCTTTGCGTTGCTCT	170	60.2
		TTTTCGGCCAGTCAGGGAAC	170	60.54
9	*LOC102173702*	ATCAAACAGAGCATAACAAACCGT	76	59.48
		CCAGGTAGTCTTGTCCGTGG	76	59.75
10	*TYR*	CCTCGGCTGATGTGGAGTTT	188	60.04
		CTGGGACATCGTTCCGTTCA	188	60.04
11	*DCT*	AGTCCTTCGCTTTGCCCTAC	176	60.04
		GACTCGGCGGTTGTAGTCAT	176	59.83
12	*ASIP*	AGCCCAGAGATGAAAGGAACC	76	59.72
		GCCACAATAGAGACAGAAGGGA	76	59.5
13	*AHCY*	GGCTGCTATGGAGGGCTATG	205	60.04
		CACCTTCTCCACAGCGTTCT	205	59.97
14	*OCA2*	ATCCTCGCTGGGGTCTACAT	117	60.11
		GCTGGGTCTGTCACCAATCA	117	59.96
15	*LOC102170144*	GCCCACGCATAGGAAGAAACT	262	60.68
		CGCAGCCGAGTTCTCAATCT	262	60.46
16	*LOC102177275*	CTCAGCATGAAAGCGTCCCT	249	60.39
		GGTCTTGTACTGGGTCAGGTG	249	60
17	*GPNMB*	CGAGCACCCTCGTCTCTATC	217	59.41
		AGGGTCTTTGCATGACTGAGG	217	60
18	*ERRFI1*	AGCGAGTTTGAGAACGGCT	242	59.63
		AGCTTCTTCAGTCCACACGC	242	60.6
19	*LRP4*	GCGAATGTGGAGGGACTCAT	121	59.82
		AGTCCGAGGTTCTGGCAAAG	121	59.96
20	*PER1*	CTTCTACGGTTCCACCACCC	143	60.04
		GACTATCTCTGTGCCCCACG	143	59.9
21	*EDN2*	CGGACAGACAGCTCCTTACG	119	60.18
		TTTGATGGCAGAAGGTGGCA	119	60.18

1) The annealing temperature.

2) The reference genes.

**Table 2:** Statistical results of the transcriptome sequencing of 18 skin samples

Breed	Sample name	Body site	Clean reads	Clean bases	GC Content	≥Q30
IMCG	701-4	#4	25411768	7580493722	51.9%	94.0%
	702-4	#4	26746318	7998380756	51.1%	93.3%
	703-4	#4	31746060	9497004254	51.3%	93.7%
	701-7	#7	27214917	8127059046	52.2%	93.8%
	702-7	#7	27556581	8222529000	52.2%	93.2%
	703-7	#7	24130965	7207794552	52.1%	93.7%
	701-14	#14	29321869	8737617176	51.1%	93.7%
	702-14	#14	26657239	7959202692	52.4%	93.2%
	703-14	#14	30270531	9042363420	51.7%	93.7%
DBG	801-4	#4	28417462	8484835900	52.3%	93.7%
	803-4	#4	29596873	8847281976	51.7%	93.7%
	805-4	#4	30288499	9049634306	52.9%	93.6%
	801-7	#7	28458954	8486181666	51.7%	93.6%
	803-7	#7	26104400	7787258936	51.5%	93.7%
	805-7	#7	29922556	8935922118	52.5%	93.2%
	801-14	#14	21885049	6523664870	51.6%	93.9%
	803-14	#14	25489640	7605321446	51.9%	93.8%
	805-14	#14	29342271	8760914902	52.2%	92.9%

GC, guanine-cytosine; IMCG, Inner Mongolia cashmere goat; DBG, Dazu black goat.

## SUPPLEMENTARY MATERIAL

**Supplement 1**. The photo of Dazu black goats (DBG, ♂), Inner Mongolia cashmere goats (IMCG, ♀), and their F1 hybrids (DBG♂ × IMCG♀).
