# Supplementary material for: Spatial variation and associated genes of total hair follicle density in goats
Source: Anim Biosci. 2025 May 12;38(7):1356–71. doi: 10.5713/ab.25.0026 (PMC12229905; doi:10.5713/ab.25.0026)
Supplement: Supplementary file 1 [file ab-25-0026-Supplementary-1.pdf]

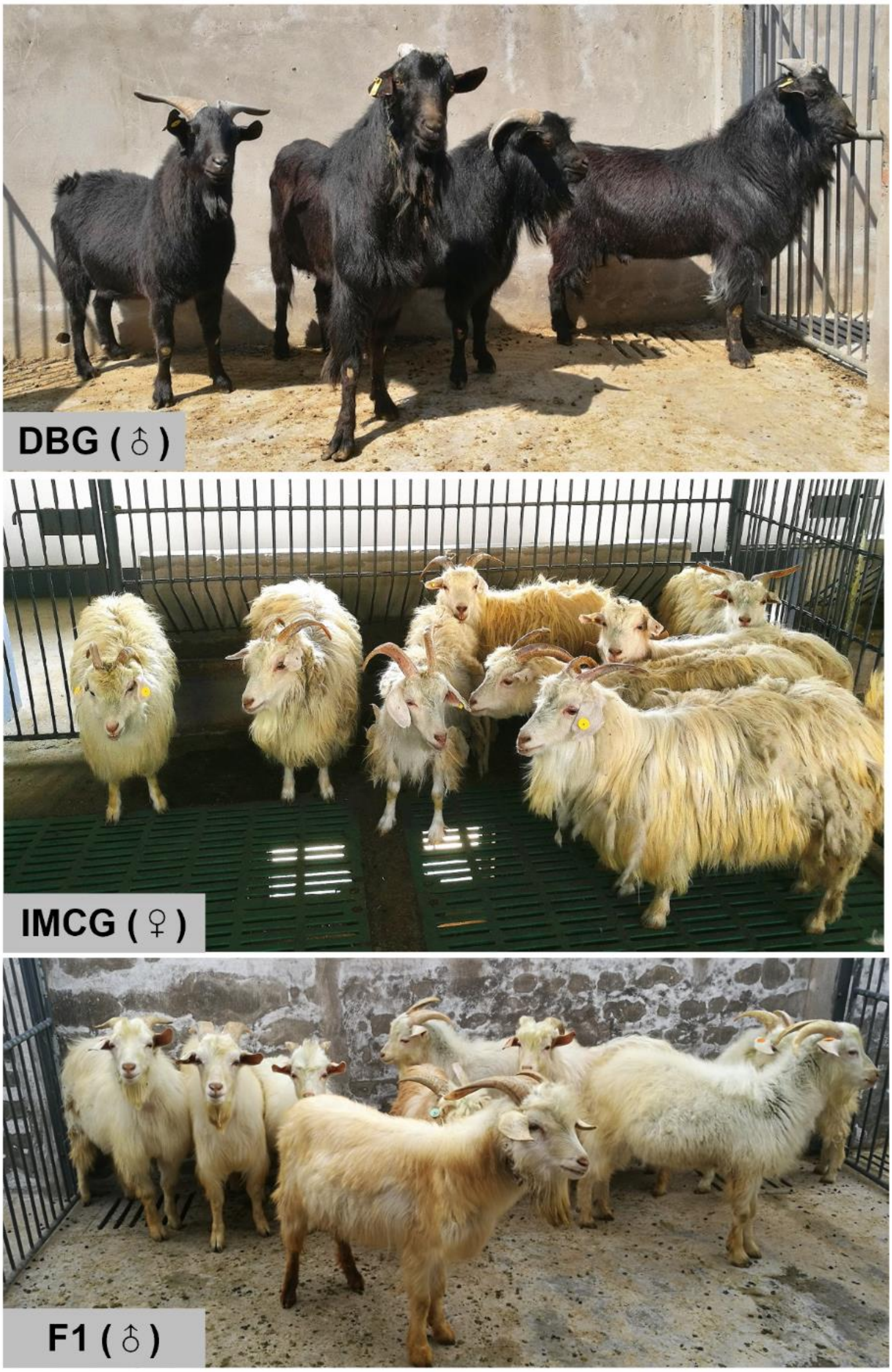

Supplement 1. The photo of Dazu black goats (DBG, ♂), Inner Mongolia cashmere goats (IMCG, ♀), and their F1 hybrids (DBG♂ × IMCG♀).
